# Respiratory pressure and flow data collection device providing a framework for closed-loop mechanical ventilation

**DOI:** 10.1016/j.ohx.2025.e00671

**Published:** 2025-06-28

**Authors:** Samuel Hastings, Jacob Mildenhall, Kayla Sinclair, Ella F.S. Guy, Jaimey A. Clifton, Jordan F. Hill, Yunpeng Su, J. Geoffrey Chase

**Affiliations:** Department of Mechanical Engineering, University of Canterbury, Christchurch, New Zealand

**Keywords:** Mechanical ventilation, Pressure, Flow, Venturi, Digital twin, Closed loop

## Abstract

This article details a pressure and flow sensor system device which enables a framework for the research and development of personalized mechanical ventilator support in a closed-loop or semi-closed-loop control system, where the measurements from this device could be hooked to digital twin models and any ventilator allowing open control.

In current practice, patient response to mechanical ventilation is highly variable. Furthermore, current weaning best-practice relies on clinical experience which can lead to variability and inequality in both care and health outcomes. Personalized care can improve these inequalities in care due to patient variability when combined with digital twin models, which simulate physiology based on patient specific data, by improving the level of care possible in the ICU (Intensive Care Unit), regardless of clinician experience and/or patient variability.

The device consists of two 3D printed custom Venturis and a Y-piece, with differential pressure sensors measuring gauge, inhalation, and exhalation pressure at the patient. The sensor system has an operating range of ±50.8 cmH_2_O and a mean error in flow data of 3.2%. The system uses BLE (Bluetooth Low Energy) communication between ESP32-S3 development boards to facilitate the closed loop framework. Within this loop, pressure data is sent to a digital beside sheet, which runs digital twin protocols and sends commands to a BLE controlled ventilator. Overall, this device allows the future development and validation of personalized mechanical ventilation treatment through integration with digital twin models.

## Graphical abstract

**Figure:**
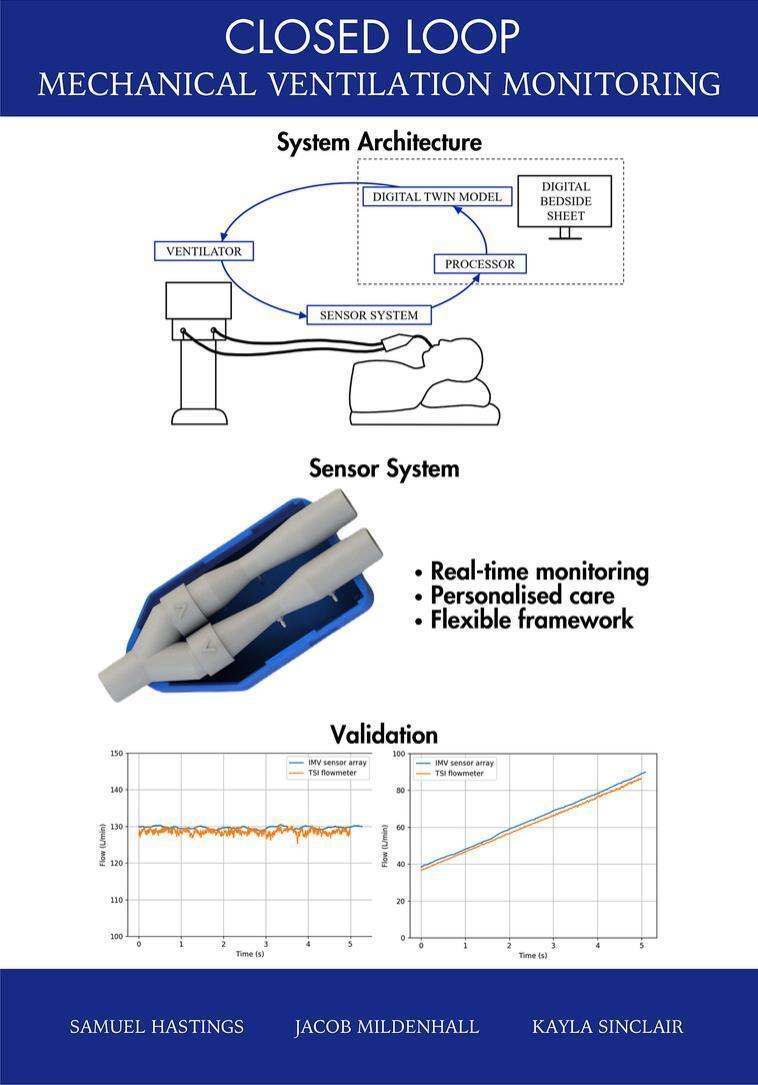

## Nomenclature

MV: Mechanical Ventilation
IMV: Invasive Mechanical Ventilation
NIMV: Non-Invasive Mechanical Ventilation
PEEP: Positive End Expiratory Pressure
PAP: Positive Airway Pressure
NZD: New Zealand Dollar
PCB: Printed Circuit Board
PLA: Polylactic Acid
STL: Stereolithography
BLE: Bluetooth Low Energy

Specifications table

**Table:** 

Hardware name	Respiratory Pressure and Flow Data Collection Device Providing a Framework for Closed-Loop Mechanical Ventilation
Subject area	Engineering and materials science
Hardware type	Measuring physical properties and in-lab sensors
Closest commercial analog	Flow sensors are commercially available but expensive (e.g., the TSI4000 which we used to validate this device).
Open source license	Creative Commons Attribution 4.0 International Public License
Cost of hardware	∼ $462 NZD
Source file repository	doi: 10.17632/pf5dbgwkbm.1
OSHWA certification UID *(OPTIONAL)*	NZ000005

### 1 Hardware in context

This work presents a set of Venturi-based flow meter and pressure sensor devices whose potential future use is demonstrated and validated in a simulated IMV breathing circuit. The device enables patient-specific respiratory modelling for control through pressure and flow data measurements and can thus be integrated with a framework to develop a closed loop MV system to convert sensor measurements into clinical metrics and recommendations using a range of digital twin or virtual patient models [[1], [2], [3], [4], [5], [6], [7], [8], [9], [10], [11], [12]].

**Fig. 1:**
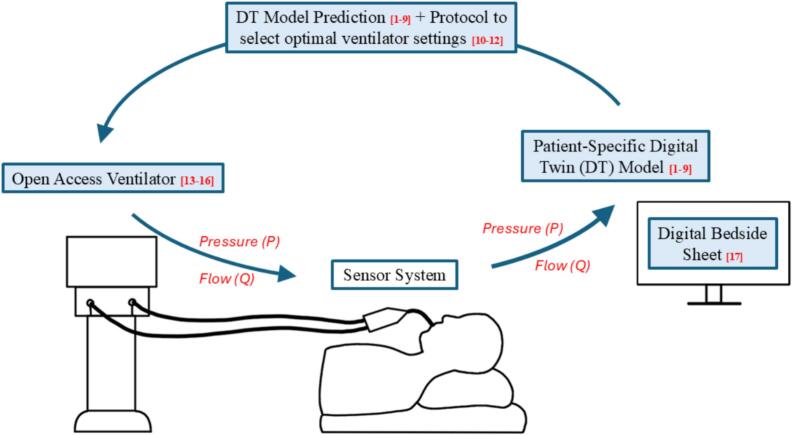
The development of a device to collect pressure and flow data can provide sensing to feed digital twin models and treatment protocols for patient-specific closed loop mechanical ventilation, based on previous research in the shaded boxes and as referenced in the figure as well.

Proof of concept has been achieved using the mePAP, one of many open-access ventilators, which is adjustable over BLE [[13], [14], [15], [16]]. The mePAP is integrated with a digital bedside sheet, developed in Microsoft Excel based on previous work by Su et al. [17]. This digital bedside sheet acts as a framework to process the data provided by this sensor apparatus for the development of personalized models. Fig. 1 shows how the device presented in this work can provide sensing to develop a closed loop MV system based on previous research, which has been further shown in a proof of concept [[1], [2], [3], [4], [5], [6], [7], [8], [9], [10], [11], [12], [13], [14], [15], [16], [17]].

The device has been adapted from previous designs for NIMV based applications [18,19]. The device was built on previous designs with internal one-way valves to reduce the system size and an equal angle Y-piece to reduce losses. Additionally, the system implements BLE transmissions and a digital bedside sheet where the data can be viewed real-time and stored.

The personalized modelling enabled by this device, and additional components, aims to solve a current clinical problem in the ICU. Patient response to MV is highly variable, and currently MV guidelines and protocols are not patient-specific [[20], [21], [22]]. There is very limited clinical consensus on care protocols for weaning patients from IMV to NIMV modalities, and eventually off ventilator support entirely [20,21]. At this time, weaning relies predominantly on clinical intuition and experience and relatively ad-hoc local guidelines or protocols which are not patient specific. The result leads to variability and inequality in care and outcomes due to patient variability and variability in clinical experience, as well as increased cost [22,23].

The device supports the development of digital twin models, which are not available with current ventilatory technology and an emerging science in their own right. These models offer the ability to accurately make patient-specific response predictions to ventilator adjustments, which enables automation of care [[1], [2], [3], [4], [5], [6], [7], [8], [9], [10], [11], [12]]. Facilitating the development of such models, as well as their application within a closed-loop system, could, in future, eliminate infrequent, invasive weaning tests and introduce non-invasive testing at a higher frequency, and a major issue in ICU medical care could be addressed, along with a range of other ventilation outcomes which might be improved [22]. Finally, the device itself differs from commercial alternatives such as the TSI4000 Flow Meter (4000 Series Analog and Digital Flow Meter, TSI, Shoreview, MN, USA) as it is significantly less expensive to produce, provides wireless communication, and is fully adjustable, with no proprietary firmware / software.

### 2 Hardware description

**Fig. 2:**
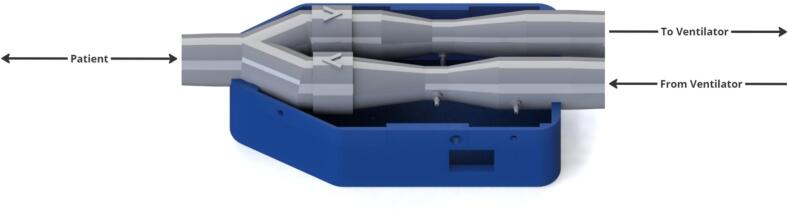
Sensor apparatus render with no electronics.

The sensor apparatus consists of two Venturis in parallel to produce individual pressure differences to measure inhalation and exhalation profiles. Flow into the Venturis is controlled by one-way valves, and a pressure sensor at the patient mouthpiece allows gauge pressure to be obtained (Fig. 2). This design is adapted from a previous design for NIVM by Guy et al. [18].

To adapt the previous design to the IMV context, the differential pressure sensors (SSCDRRN020NDAA5) were updated to reflect the lower flows measured in IMV. The new sensors offer a lower full-scale span, with the same percentage error as the previous sensors which results in improved data resolution. Data quality has also been improved by the introduction of symmetric Y-piece. Previously, an asymmetric Y-piece produced high quality exhalation data at the cost of poorer inhalation data. This device offers equally high data quality for inhalation and exhalation flows, making it more suitable for hysteresis analysis of Pressure-Volume loops.

The Venturis were designed to be integrated in series with standard IMV circuitry, using standard 22  mm male and female connections [24]. One-way valves have been integrated into the Venturis, removing the need for an added modular component as seen in the previous design by Guy et al [18]. Furthermore, a housing has been created to isolate electrical components and keep the device compact. The housing and software was designed with support for a future modular rapid expiratory occlusion (REO) attachment [18]. Custom PCBs have been created to incorporate the ESP32-S3 development boards into the system.

To summarize, this device is designed for pressure and flow data collection, using two Venturis in parallel, for invasive mechanical ventilation research. This device facilitates the development and testing of patient-specific respiratory models, and provides researchers with:

- A low-cost sensor apparatus with comparable performance to commercial alternatives.
- Wireless communication between sensor and processer, facilitating data collection.
- A specialized sensor design for IMV data.
- High level of customization for both firmware and hardware to adapt to any data processing framework or ventilator circuitry.

### 3 Design files

CAD files: The physical design was performed in SolidWorks 2023 [25].

3D printing: All 3D printing was done in PLA on Bambu Lab printers.

Electronics: PCBs were designed in Altium [26]. These were manufactured by JLCPCB and populated in the SMT lab at the University of Canterbury.

Software and firmware: The code for the sensor system device and the digital bedside sheet ESP32s were written in C, Arduino, and C++ using Visual Studio Code [27].

### 4 Design files summary

All the design files are available at doi: 10.17632/pf5dbgwkbm.1. Table 1 outlines file locations.

**Table 1:** Design file summary.

**Design file name**	**File type**	**Open-source license**	**Location of the file**
Barb	*CAD (SLDPRT) and STL files*	CC BY 4.0	Device, Hardware, 3D Printed Components
Base Plate	*CAD (SLDPRT) and STL files*	CC BY 4.0	Device, Hardware, 3D Printed Components
Housing Base	*CAD (SLDPRT) and STL files*	CC BY 4.0	Device, Hardware, 3D Printed Components
Housing Top	*CAD (SLDPRT) and STL files*	CC BY 4.0	Device, Hardware, 3D Printed Components
One Way Valve	*CAD (SLDPRT) and STL files*	CC BY 4.0	Device, Hardware, 3D Printed Components
Venturi Exhalation	*CAD (SLDPRT) and STL files*	CC BY 4.0	Device, Hardware, 3D Printed Components
Venturi Inhalation	*CAD (SLDPRT) and STL files*	CC BY 4.0	Device, Hardware, 3D Printed Components
Y-Piece	*CAD (SLDPRT) and STL files*	CC BY 4.0	Device, Hardware, 3D Printed Components
Control Board	*Altium files*	CC BY 4.0	Device, Hardware, PCBs, ControlBoard
Sensor Board	*Altium files*	CC BY 4.0	Device, Hardware, PCBs, SensorBoard
Gauge Board [9]	*KiCAD files*	CC BY 4.0	Device, Hardware, PCBs, GaugeBoard
Sensor System Code	*.ino,.cpp,.h files*	CC BY 4.0	Device, Software, Code, SensorSystem
Digital Bedside Sheet ESP32 Code	*.ino,.cpp,.h files*	CC BY 4.0	Device, Software, Code, DBS
Modified mePAP code	*.c,.h files*	CC BY 4.0	Device, Software, Code, mePAPClient
Digital Bedside Sheet Excel Spreadsheet	*.xlsm file*	CC BY 4.0	Device, Software

### 5 Bill of materials summary

The completed bill of materials is attached in an Excel spreadsheet (*doi: 10.17632/pf5dbgwkbm.1**)*. This spreadsheet outlines components and their cost for the base device (∼NZ$462).

### 6 Build instructions

1. Place an order for all components listed on the bill of materials.
2. Populate the PCBs in accordance with the Altium project files. To populate the PCBs solder on the *100 nF Capacitor*, *2 k7 Resistors, 4 k7 Resistors*, headers (*3PinMolexKKHdr, 4PinMolexKKHdr, 3POS MOLEX Female Header, 4POS MOLEX Female Header*), and sensors (*SSCDRRN001PD2A5 Pressure Sensor, SSCDRRN020NDAA5 Pressure Sensor*). Solder *Socket Headers* into the through holes for the ESP32, before mounting it. The populated *Control Board*, *Gauge Board* (Guy et al. 2023) and *Sensor Boards* are shown in Fig. 3 [18].

**Fig. 3:**
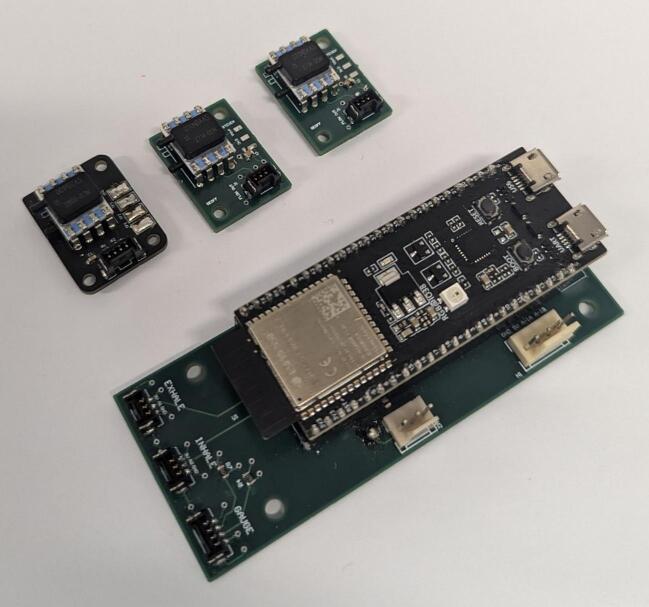
Populated printed circuit boards.
3. Wire the *Switch* and *3PinMOLEXKKConn.* If a switch with LED is not available, a basic switch, LED*,*_resistor*,* and *3PinMOLEXKKConn* can be wired together as shown in Fig. 4.

**Fig. 4:**
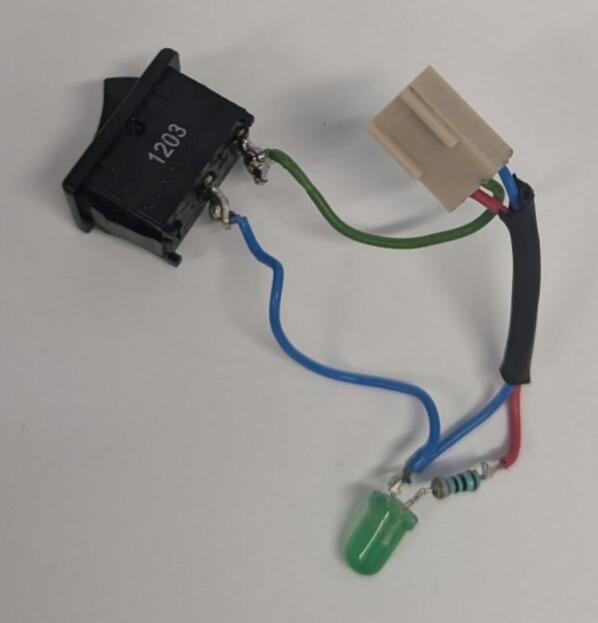
Assembly of switch and LED with 3 pin MOLEX connector.
4. Print the *Housing Top* and *Housing Base* on the same print plate orientated as shown in Fig. 5. The parts were printed in PLA on a BambuLab Printer (BambuLab, TX, USA) with automatic tree support and an extra fine layer height of 0.08 mm. All other 3D printed parts should be printed on a separate plate as shown in Fig. 6, with normal support and a layer height of 0.12 mm.

**Fig. 5:**
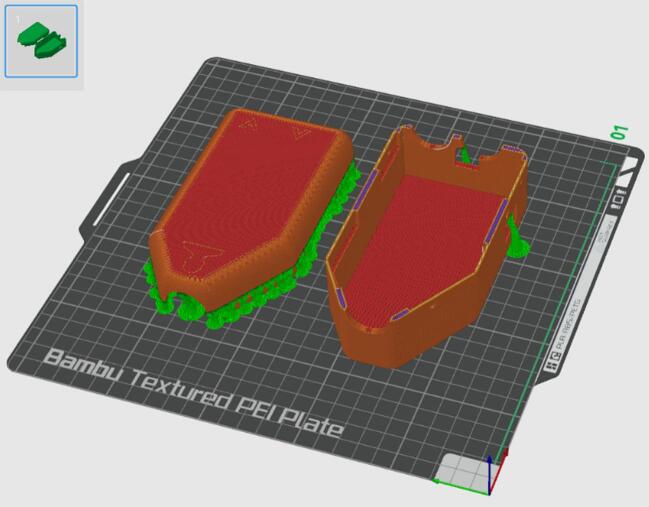
Print orientation for the external housing of the device (orange/red) with support in green and blue. (For interpretation of the references to colour in this figure legend, the reader is referred to the web version of this article.)

**Fig. 6:**
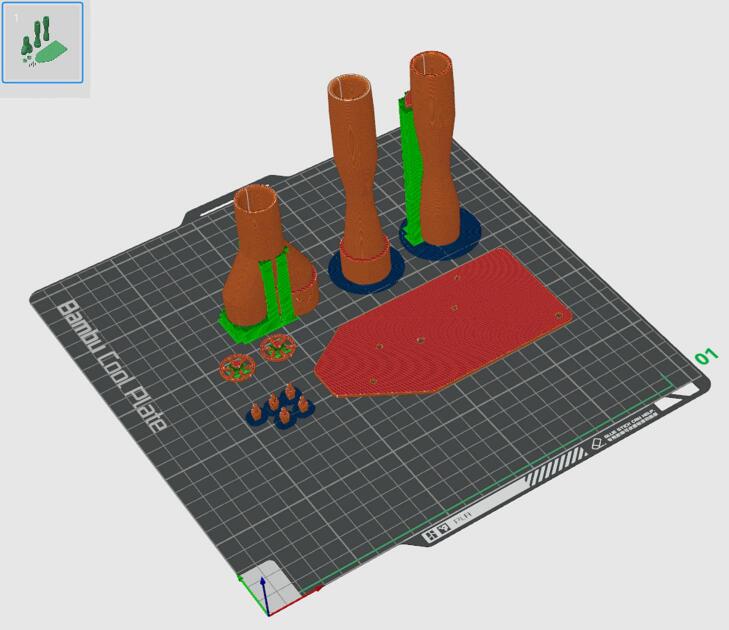
Print orientation for the internal components of the device (orange/red) with support in green and blue. (For interpretation of the references to colour in this figure legend, the reader is referred to the web version of this article.)
5. Remove support material from components and attach barbs to Venturis and Y-piece using superglue (Fig. 7). Remove the *IPN913948 Membrane* from Hudson RCI One-Way Valve and attach to 3D printed *One Way Valve*. Insert into *Y-piece* and *Inhalation Venturi* with membrane facing out (Fig. 8). Tap the four small holes on each of *Housing Top* and *Housing Base* with a M3 × 0.5 mm tap.

**Fig. 7:**
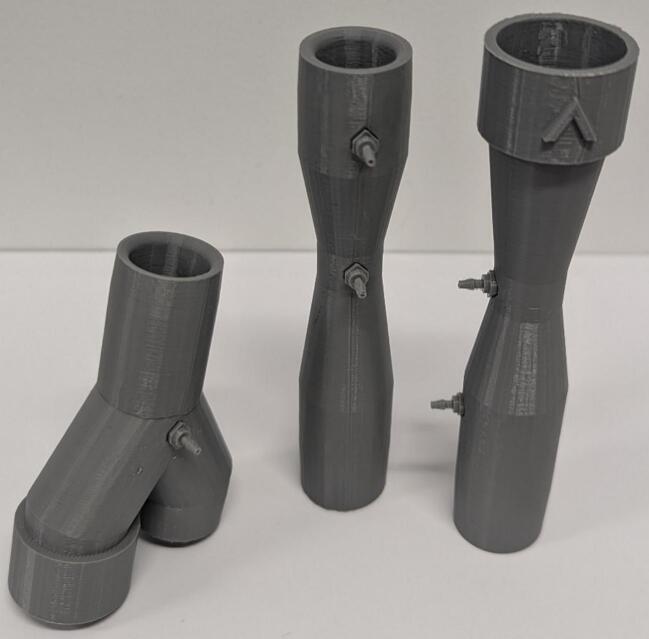
Barbs attached using superglue.

**Fig. 8:**
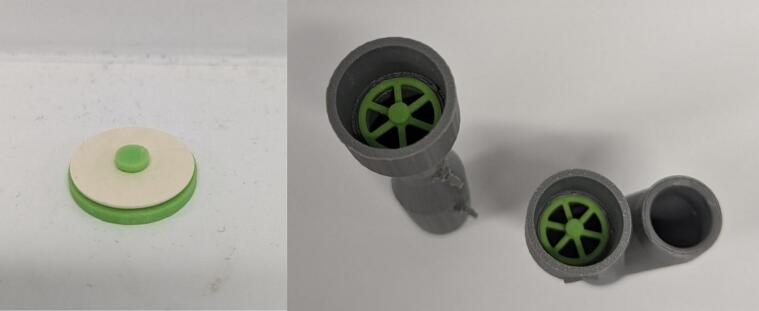
One-way valves with membrane (left) and inserted into Venturi and Y-piece with membrane removed for visual clarity (right).
6. Connect inhalation and exhalation Venturis as shown in Fig. 9. When placed on a flat surface, the inhalation barbs should be parallel to the surface. The exhalation barbs should be at a 45° angle down from the inhalation barbs and the Y-piece barb should face directly into the surface.

**Fig. 9:**
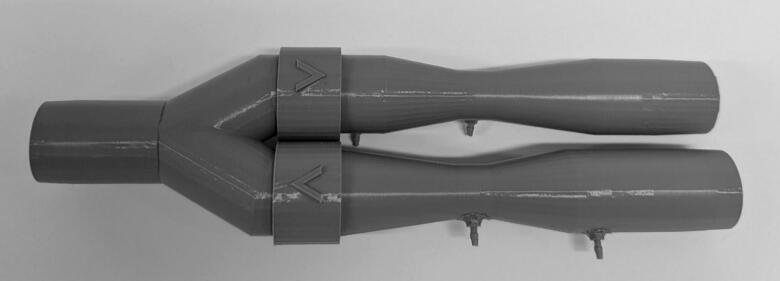
Connected Inhalation Venturi, Exhalation Venturi and Y-piece.
7. Connect the inhale and exhale *Sensor Board*s to the *Control Board* using *3-pin connectors*. Connect the *Gauge Board* to the *Control Board* using the *4-pin connector*.
8. Attach a short length of *Silicone Tubing* to the sensor ports. The lengths are dependent on the positioning of each sensor and should be adjusted to suit. Each sensor has two ports, one that sits higher than the other. The higher port on each *Sensor Board* sensor should be connected to the inlet barb of their respective Venturi. The higher port of the *Gauge Board* sensor should be connected to the Y-piece barb. The lower port of each *Sensor Board* sensor should connect to the throat barb of their respective Venturi. The lower port on the *Gauge Board* sensor should be left disconnected. Fig. 10 shows the connection of all components within the device, spaced out for clarity.

**Fig. 10:**
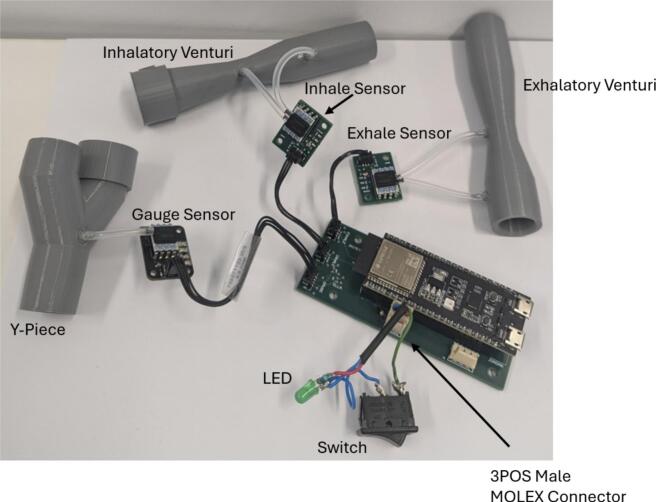
Assembled circuit, spaced out for clarity.
9. Attach the *Gauge Board* to the *Base Plate* using M2 screws (Fig. 11). Attach *Control Board* to the *Base Plate* with M3 screws. Leave the inhale and exhale *Sensor Boards* loose but arrange them as shown in Fig. 12. Do not attach the switch circuit at this stage.

**Fig. 11:**
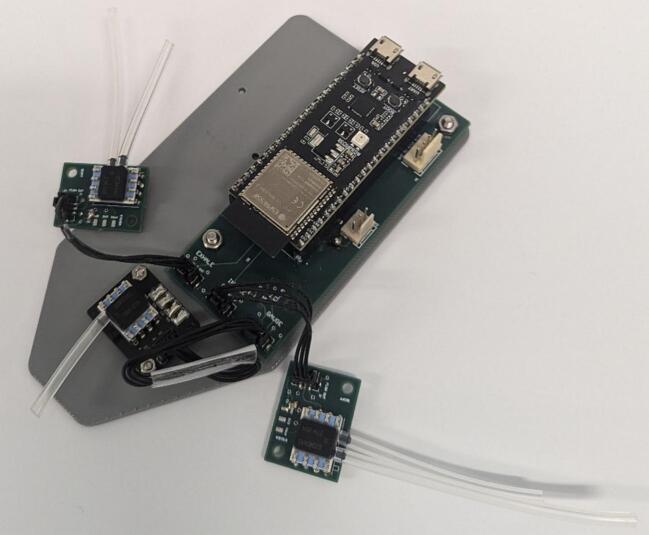
Base plate with internal electrical components attached.

**Fig. 12:**
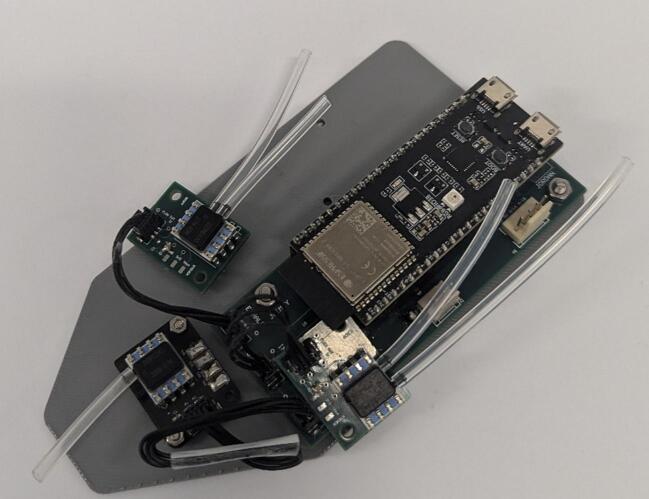
Internal components attached to base plate with inhale and exhale Sensor PCBs arranged.
10. Slide *Base Plate* into *Housing Base* and connect the switch circuit (see Fig 13). Align *Inhalation Venturi*, *Exhalation Venturi* and *Y-Piece* above the system and connect the end of the silicone tube to the free barbs (see Fig. 14).

**Fig. 13:**
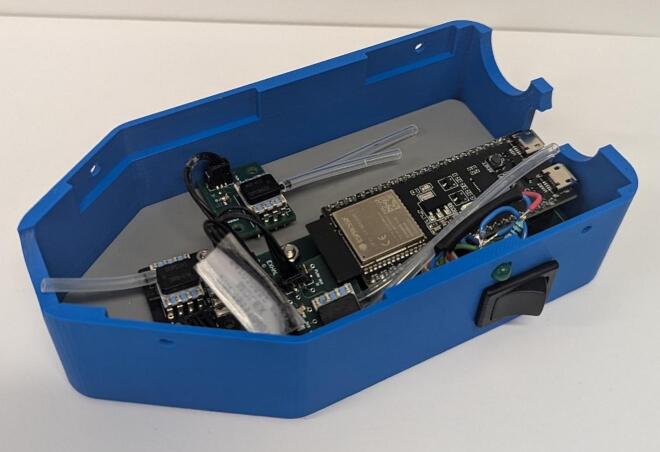
Base Plate and internal electrical components in Housing Base.

**Fig. 14:**
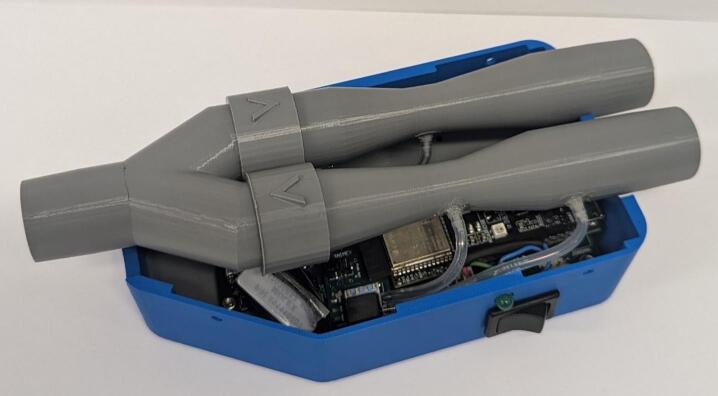
Venturis and Y-piece aligned with Base Housing and sensor port connected.
11. Attach *Housing Top* to *Housing Base* and insert 4 M3 grub screws into the tapped holes to secure the external housing shut (Fig. 15).

**Fig. 15:**
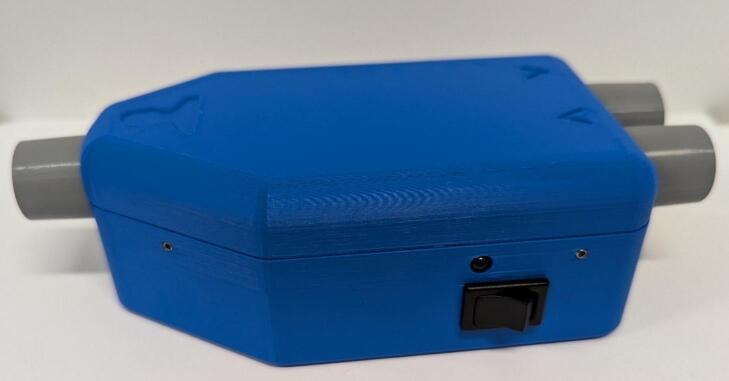
Fully assembled device.

### 7 Operation instructions

1. Connect the sensor system to any source of power via the micro-USB connector and turn it on. The power LED should be on.
2. Plug the digital bedside sheet ESP32-S3 development board into a computer with the digital bedside sheet Excel document downloaded.
3. Open the Excel digital bedside sheet document, enabling macros when prompted.
4. Navigate to file – options – add-ins. Toggle “Manage: COM Add-ins” and select “Go…”. Check Microsoft Data Streamer for Excel and click OK (Fig. 16).

**Fig. 16:**
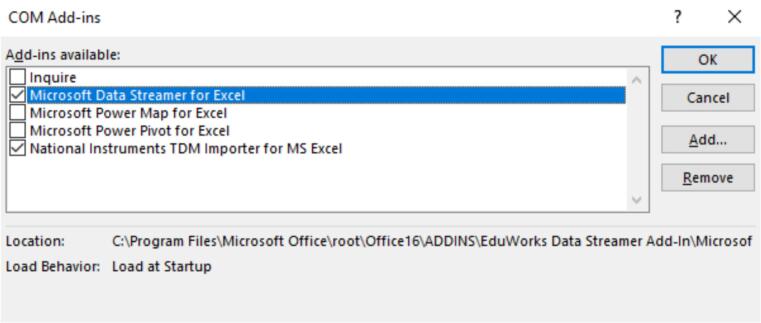
Data streamer toggle.
5. Navigate to Data Streamer on the Excel taskbar. Select connect a device and select the COM port corresponding to the ESP32-S3 development board for the digital bedside sheet. Note that if the digital bedside sheet board is ever reset, this step will need to be repeated.
6. If the mePAP is being used, power it via both its micro-USB connector and its wall power supply. To simulate basic IMV functionality on the mePAP, the provided code can be modified to raise and lower the desired pressure with consistent timing, providing a controlled pressure flow profile. A PEEP valve can also be added to the expiratory Venturi of the device to mirror the PEEP valves found within an invasive mechanical ventilator. If further sensing or flow control is provided, it is recommended that an invasive mechanical ventilator is used. It is not recommended that a controlled volume flow profile is used as the digital bedside sheet will only output a desired pressure for ventilation.
If the mePAP is not being used, connect to the ESP32-S3 development board for the digital bedside sheet with any extra device. The free nRF connect application is suggested. Alternatively, edit line 59 in Software/Code/DBS/inc/BLEControl.h to set the number of devices that the digital bedside sheet board is expecting to one. This change produces a human-in-the-loop system, where the digital bedside sheet will display a pressure for the user to input into a ventilator.
7. Connect the chosen ventilator to the sensor system using the relevant respiratory circuitry.
8. Select “Start Data” in Data Streamer in the Excel taskbar. Observe the *Data In* sheet to see the status of the system. If “start advertising” appears, not all devices are connected. It is advised to restart the suspected device to force reconnection. If numbers are appearing, all devices are connected.
9. When all devices are connected, navigate to the *User Interface* sheet. Confirm that the sensor system is not calibrating (Fig. 17). If the sensor system is finished calibrating, connect the chosen ventilator (mePAP or an invasive mechanical ventilator) and begin monitoring. It is crucial to not turn on the ventilator while the sensor system is zeroing. Zeroing should take no longer than 10 s. If the sensor system is turned off, it will need to re-zero when it is turned on again.

**Fig. 17:**
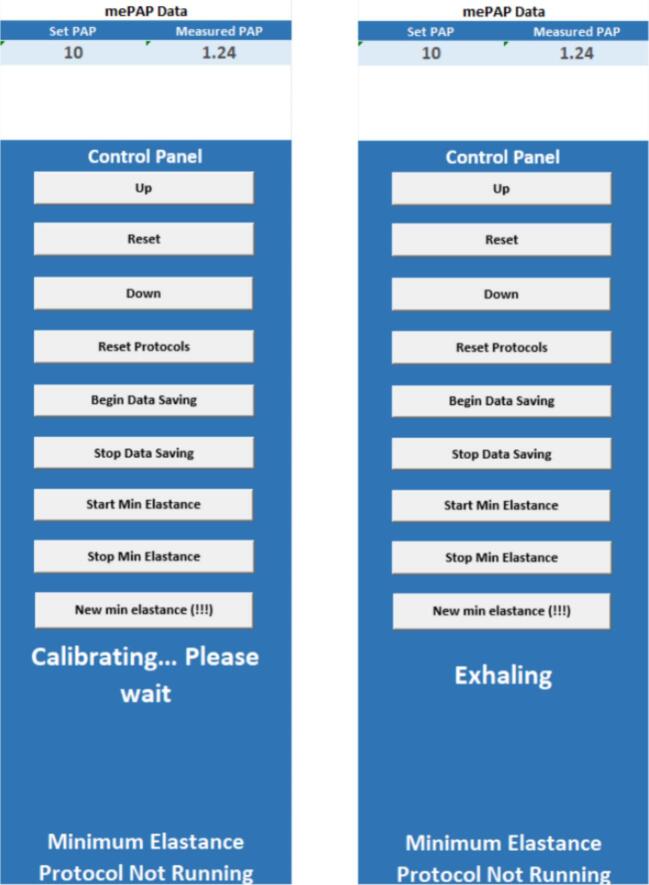
User interface ready for measurement (left) and calibrating (right).
10. Protocols for minimum elastance, kink detection and apnoea detection can be turned on via buttons in the digital bedside sheet. Note that minimum elastance will work only with the mePAP as closed loop control of a ventilator is required. Additional data analysis can be implemented via Excel VBA. The Excel VBA coding interface can be accessed via the *alt + F11* shortcut. Finally, it is advised that the file path in the *Write CSV* Excel VBA code is changed to a suitable file path.
11. Turn on the chosen ventilator and view plots and run protocol via the *User Interface* sheet (Fig. 18).

**Fig. 18:**
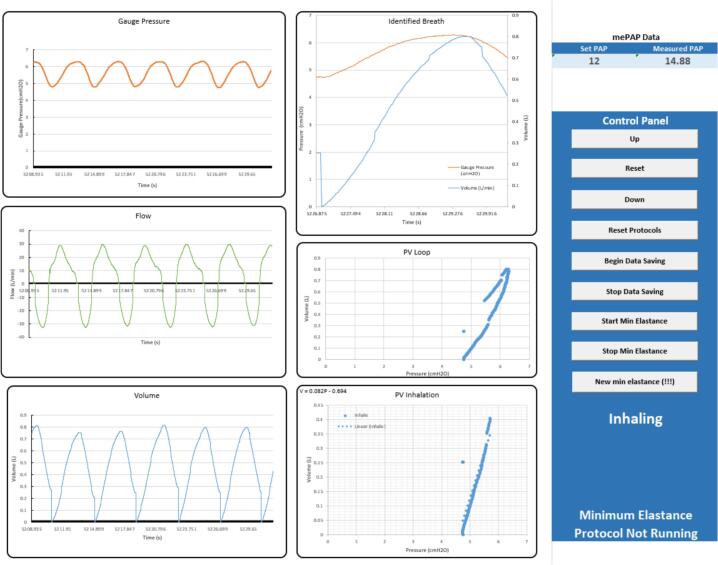
User Interface sheet.

### 8 Validation and characterization

The device is intended to be used to facilitate closed-loop invasive mechanical ventilation in an ICU setting. The digital bedside sheet operates as a framework for digital twin protocols to be implemented, which require the patient specific metrics monitored by the device. The device has the following relevant operating parameters:

- The sensor system device and peripheral digital bedside sheet operate with a 5V supply.
- The pressure sensor operating range limits the use case to ±50.8 cmH_2_O.
- The Venturi design and pressure sensor operating range limit the flow (Q) measurements to 222L/min (by Eq. (1), given a discharge coefficient *c_D_*=0.97, and air density of *ρ* = 1.293kg/*m*^3^, where Δ*P* is change in pressure (Pa) across a Venturi inlet and throat, A_1_ is the cross-sectional surface area of the Venturi inlet (m^2^) and A_2_ is the cross-sectional surface area of the Venturi throat (m^2^)).
- The datatype used to store runtime limits how long the device can operate continuously to 2^32^*ms* (49 days).
- Data frequency is limited by the BLE transmission rate to 160*Hz*.

(1) $$\begin{array}{c} Q=c_{D}A_{2}\sqrt{\frac{2\Delta P}{\rho \left(1-{\left(\frac{A_{2}}{A_{1}}\right)}^{2}\right)}} \end{array}$$

**Fig. 19:**
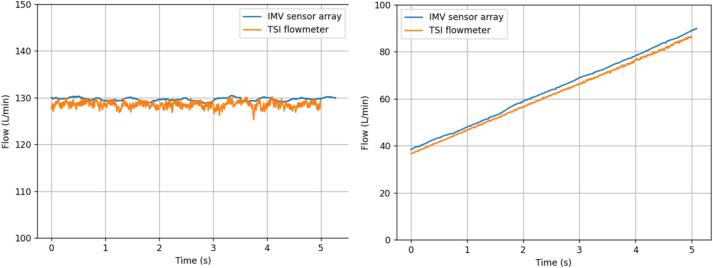
IMV sensor array and TSI flowmeter comparison with constant 20 cmH_2_O PAP (left) and increasing PAP (right).

**Fig. 20:**
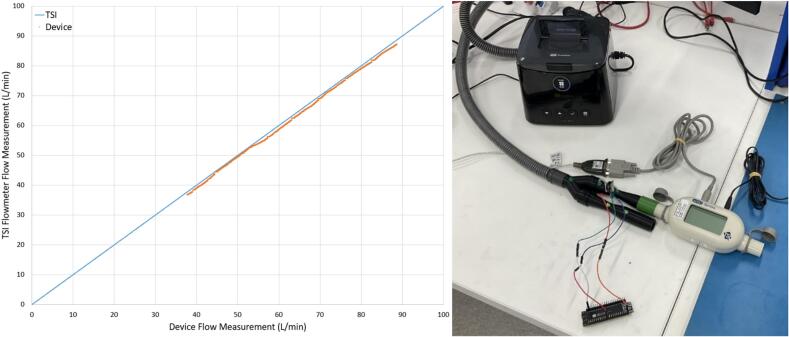
Validation of device flow against TSI flowmeter using CPAP through test setup vented to atmosphere (right).

The device was validated against a TSI4000 Flow meter (4000 Series Analog and Digital Flow Meter, TSI, Shoreview, MN, USA) using flows generated by a Fisher and Paykel Healthcare SleepStyle CPAP Machine (SleepStyle SPSCAA, Fisher and Paykel Healthcare, East Tamaki, Auckland, NZ). The exhalation Venturi was connected in series with the TSI flowmeter which was vented to atmosphere. The flows measured by the IMV sensor system and the TSI flowmeter were compared at both a constant PAP and a linearly increasing PAP (Fig. 19). The increasing PAP test was conducted by measuring the flow through the sensor array during the ramp period of the CPAP machine. The constant PAP test correlated well with the TSI measured flows, yielding a mean 0.82 % percentage error. The increasing PAP test yielded a mean 3.2 % percentage error compared to the TSI flowmeter. The error could be a result of resistive losses over the Venturi or leakage through the adapter (Fig. 20).

To validate the ability for this system to work within the closed loop system, a simple minimum elastance protocol was implemented. The minimum elastance protocol was tested on a mechanical lung which was ventilated by the mePAP. The varying elastance expected in a real patient ventilation response was simulated by shifting a spring on the mechanical lung. A mechanical lung was used to demonstrate the ability to gather data and close the loop in a lab setting to ensure a known ground truth in providing the technology validation necessary for ethics approval and testing on human subjects. No human testing would be possible before bench test validation. A PEEP valve was also added to the expiratory Venturi of the device to simulate the PEEP valve found inside an invasive mechanical ventilator.

Air flow data measured by the system was used to calculate the elastance of the mechanical lung at each breath by taking the reciprocal gradient of the portion of the measured pressure–volume loop representing inhalation (Eq. (2)).

(2) $$\begin{array}{c} E=\frac{\Delta P_{\mathrm{inhale}}}{\Delta V_{\mathrm{inhale}}} \end{array}$$

**Fig. 21:**
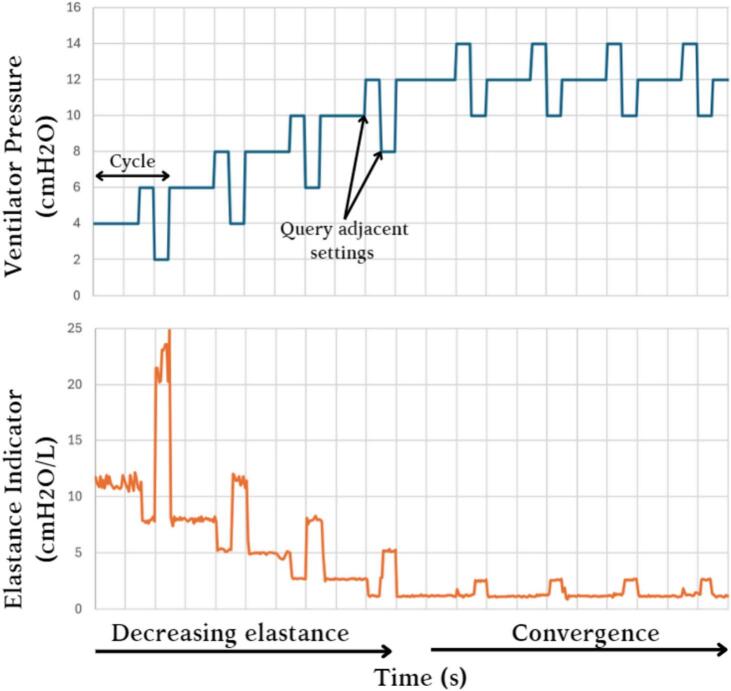
Convergence of minimum elastance protocol.

Where *E* is elastance (cm H_2_O/L), Δ*P_inhale_* is the change in pressure in inhalation (cm H_2_O) and Δ*V_inhale_* is the change in volume in inhalation (L). Pressure settings within a range of ±2 cm H_2_O were queried across a cycle to observe the optimal pressure setting for minimum elastance. After each cycle, the pressure setting was automatically adjusted to correspond to the measured minimum elastance, via a BLE command to the mePAP. Convergence was achieved across a range of 8 cm H_2_O and a timeframe of approximately 30 min (Fig. 21).

### Ethics statement

No human or animal testing was conducted in the development of this device, and no ethics approval was required for testing in a lab setting.

### CRediT authorship contribution statement

**Samuel Hastings:** Writing – review & editing, Writing – original draft, Visualization, Validation, Software, Methodology, Investigation, Conceptualization. **Jacob Mildenhall:** Writing – review & editing, Writing – original draft, Visualization, Validation, Software, Methodology, Investigation, Conceptualization. **Kayla Sinclair:** Writing – review & editing, Writing – original draft, Visualization, Validation, Software, Methodology, Investigation, Conceptualization. **Ella F.S. Guy:** Writing – review & editing, Supervision. **Jaimey A. Clifton:** Writing – review & editing, Supervision. **Jordan F. Hill:** Writing – review & editing, Supervision. **Yunpeng Su:** Writing – review & editing, Supervision. **J. Geoffrey Chase:** Writing – review & editing, Supervision.

## Declaration of competing interest

The authors declare that they have no known competing financial interests or personal relationships that could have appeared to influence the work reported in this paper.
